# Simultaneous laparoscopic cholecystectomy and removal of an intrauterine device translocated to the right subdiaphragmal region: a case report

**DOI:** 10.4076/1757-1626-2-6198

**Published:** 2009-08-25

**Authors:** Salih Krasniqi, Elvis Ahmeti, Sejdullah A Hoxha, Halit Ymeri, Ismet Shaqiri, Nexhmije B Kastrati-Spahija, Avdyl S Krasniqi

**Affiliations:** 1 Department of Surgery, University Clinical Centre of KosovaRrethi Spitalit str. p.n., Prishtina, 10000Republic of Kosovo; 2 Department of Radiology, University Clinical Centre of KosovaRrethi Spitalit str. p.n., Prishtina, 10000Republic of Kosovo; 3 Department of Gynecology and Obstetrics, University Clinical Centre of KosovaRrethi Spitalit str. p.n., Prishtina, 10000Republic of Kosovo

## Abstract

**Introduction:**

Intrauterine devices are often accompanied by various complications, of which the uterine perforation constitutes the most dangerous one.

**Case presentation:**

We present a case of a 41-year-old woman complaining of right upper quadrant pain. She had an intrauterine device inserted 12 years earlier without regular follow-up. Abdominal plain X-ray revealed the intrauterine device trans-located into the right subdiaphragmal area. Abdominal ultrasound showed gallbladder stones without any other sonographic pathologic finding. Patient underwent simultaneous laparoscopic cholecystectomy and removal of the intrauterine device from the right subdiaphragmal area.

**Conclusion:**

Laparoscopy is an appropriate method for removal of intrauterine device translocated to the right subdiaphragmatic region.

## Introduction

The use of laparoscopy enables close inspection of inner organs. Among the advantages of the laparoscopy are its use in trauma setting, blunt and/or penetrating trauma, acute abdomen and peritonitis [1,2]. Use of intrauterine devices (IUD) has been found to be associated with several complications such as bleeding, perforation or migration into surrounding tissues or the omentum. For dislodged IUDs the removal is recommended because of the potential inflammatory responses that may cause obstruction or perforation [3]. Here we present a case of patient that had simultaneous laparoscopic removal of dislodged IUD and the gallbladder.

## Case presentation

**Figure 1.**
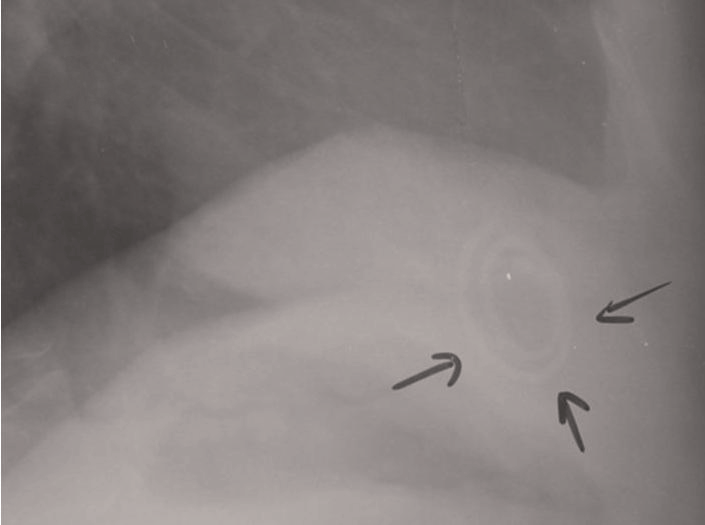
Chest X-ray PA showing the radioopaque IUD in the right subdiaphragmal region.

**Figure 2.**
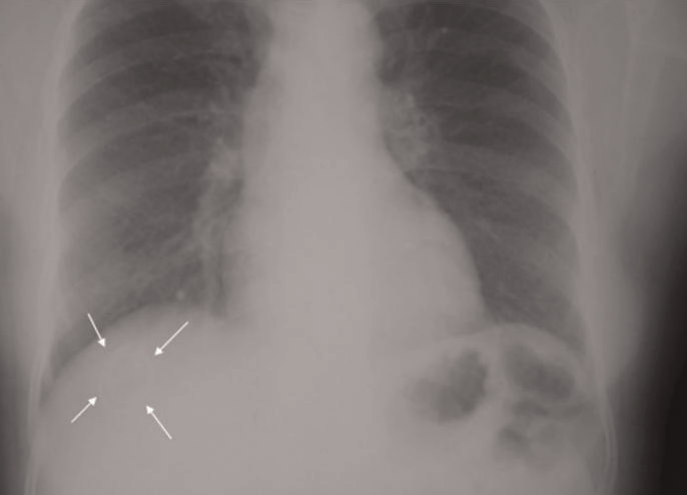
Chest X-ray LL showing the radioopaque IUD in the right subdiaphragmal region.

**Figure 3.**
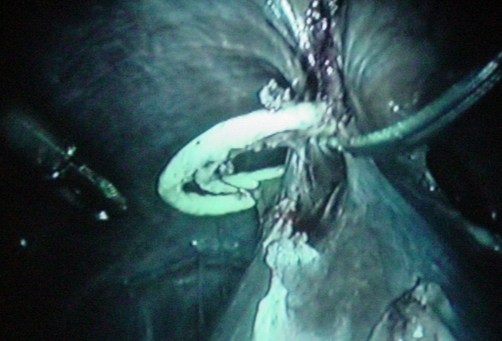
The IUD “in situ” during laparoscopy.

**Figure 4.**
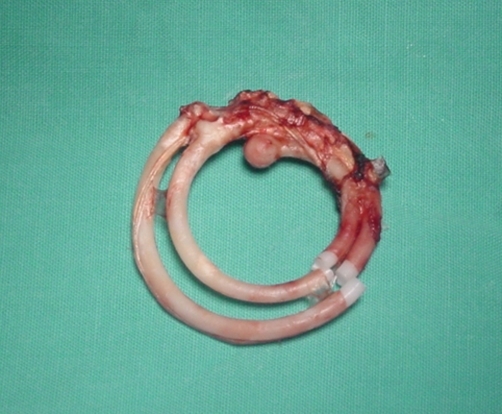
The IUD externalized.

A 41-year-old Kosovan Albanian woman presented with right upper quadrant (RUQ) pain for six months. The past medical history revealed the insertion of an intra uterine device (IUD) 12 years ago without regular follow-up. A chest X-ray showed a radio opaque foreign body resembling IUD in the right subdiafragmal area (Figures 1 and 2). The abdominal ultrasound showed only presence of gallbladder stones. The biochemistry and hematology tests were within normal range. The patient was electively taken to the operating room. 4-port “Wolf” laparoscopic equipment was used to remove the translocated IUD (Figures 3 and 4) and the gallbladder as well. The patient was discharged after 24 hours in excellent condition.

## Discussion

Since the first reported laparoscopy in 1901 - “ventroscopy” and until now the indications for this method of examination has been constantly increasing [1]. The first cases of removal of dislodged IUDs using the laparoscopy have been published in late 70’s [4,5]. Dislodgement of the IUD is accompanied with different complications. One of the most frequent is unwanted pregnancy, whether intrauterine or ectopic [6-10].

As it has been already stated the dislodgment can bring the IUD in the surrounding organs or the omentum. There were reported cases of the dislodgment in small intestines, rectosigmoid colon, peritoneum, gallbladder, appendix, annexes, iliac vein as well as the omentum [3-20]. By carefully searching the literature published so far we found 44 reported cases of the IUD displaced in rectum and/or sigmoid. In eight of them, the removal of the displaced IUD was performed transrectally [8]. These cases emphasize the importance of the rectal and endoscopic examination in patients suspected or found to have dislodged IUD [6-13]. The most frequent anchorage of these dislodged devices was the omentum (45 cases) and peritoneum (41 cases). There are reported cases of the localization in periappendicular area (9 cases) and in the small intestines (3 cases) [9,14]. In 24 reported cases the dislodgement of the IUD was in the urinary bladder and one of them was accompanied with stone formation in the bladder around the IUD [9,15,16]. To our knowledge, the case we present is the only reported IUD dislodgement in the upper abdomen, or more specifically in the right subdiaphragmal area.

Due to a potential inflammatory response and consequent obstruction and/or perforation, the majority of authors recommend the removal of the dislodged IUD laparoscopically. Laparotomy remains an option in cases were laparoscopy may not be successful [17,20]. Driven by these recommendations we decided to use laparoscopy for the removal of this dislodged foreign body, and at the same time perform the cholecystectomy. 24 hours later the patient was discharged from the hospital in excellent condition. To our awareness, this is the only reported case of the laparoscopic concurrent removal of a foreign body and cholecystectomy.

In conclusion, we have found the laparoscopy as a method of choice for the removal of the dislodged IUD because of the patients’ comfort and minimal hospital stay.

## Abbreviations

IUD: intrauterine device
RUQ: right upper quadrant
